# Recombinant DyP peroxidases for green synthesis and characterization of poly(pyrogallol)

**DOI:** 10.1038/s41598-026-64069-8

**Published:** 2026-08-05

**Authors:** Marina Refaat, Khaled O. Sebakhy, Menna-t-Allah M. Saad, Marwa ElRakaiby, Mohamed H. Habib, Maha M. Ismail

**Affiliations:** 1 Department of Microbiology and Immunology, Faculty of Pharmacy, Cairo University, Kasr El-Aini, Cairo, 11562 Egypt; 2 Chemical and Industrial Processes Engineering, Faculty of Engineering, Bahrain Polytechnic, Isa Town, Kingdom of Bahrain; 3 Department of Microbiology and Immunology, Faculty of Pharmacy, Egypt-Japan University of Science and Technology (EJUST), New Borg El-Arab City, 21934 Alexandria Egypt

**Keywords:** Pyrogallol, DyP-peroxidases, Green polymerization, Conjugated polymers, Polymer characterization, Biochemistry, Chemistry, Materials science

## Abstract

Phenolic compounds pose a major threat to water pollution due to their high toxicity. Enzymes have emerged over recent years to act as a green alternative for cleaning up and utilizing phenolic compounds in nature. DyP peroxidases, a member of the oxidoreductase family, have shown promising ability to convert dyes into harmless compounds and have been found indispensable tools for green polymerization. In this study, three recombinant DyP peroxidases were used to convert pyrogallol (PG) into a polymer which upon characterization using Fourier-transform infrared spectroscopy (FT-IR), Raman spectroscopy, powder X-ray diffraction (XRD) and scanning electron microscopy (SEM) have revealed intricate details characteristic of poly(pyrogallol) (PPG). The synthesized polymer showed a rigid structure, with potential use in material science applications such as coatings or binding agents, as well as broader aspects in environmental and biomedical research.

**Supplementary Information:**

The online version contains supplementary material available at 10.1038/s41598-026-64069-8.

## Introduction

Water pollution arises from multiple sources, with the most significant being the release of untreated sewage and agricultural runoff^1^. These sources introduce a complex mix of pollutants into water bodies, including animal waste, fertilizers, pesticides, suspended particles, and industrial organic compounds such as phenolic substances^2^. As a result, the quality of water in rivers and streams is greatly compromised. Phenolic compounds are especially problematic because they are toxic, resistant to degradation, and can pose serious risks to both aquatic organisms and human health^3^. The ongoing discharge of untreated or insufficiently treated wastewater remains a major environmental issue, emphasizing the urgent need for efficient treatment methods^4^.

Biological polymerization systems offer clear advantages over traditional chemical methods, particularly in selectivity, sustainability, and reaction conditions. Peroxidases have found application in beneficial polymerization reactions playing a role in the conversion of lactones and other cyclic compounds into polymers useful for industrial applications^5^. Enzymes such as DyP-type peroxidases can catalyze reactions with high precision, enabling the formation of polymers with more controlled structures than unwanted side products. Unlike chemical polymerization, which often relies on high temperatures, strong acids or bases, and potentially harmful solvents, enzymatic processes usually proceed under milder, and safer conditions^6^. This not only reduces energy consumption but also makes the process environmentally friendly. In addition, biological systems support green chemistry approaches by using renewable catalysts and producing fewer toxic byproducts^7^. Additionally, enzymatic catalysis can provide better control over polymer architecture, including molecular weight distribution and functional group placement, which is often difficult to achieve using traditional chemical methods without complex modifications^8^.

In recent years, enzymes have emerged as promising tools in wastewater treatment due to their ability to transform toxic compounds into mild and environmentally friendly products^9^. In addition to degradation, certain enzymes such as DyP-type peroxidases and laccases can catalyze the polymerization of phenolic compounds, converting them into higher-molecular-weight polymers that are less soluble and less toxic, and can be easily removed from wastewater^10^. In a recent study, Refaat and coworkers were able to polymerize beta-naphthol using a recombinant laccase. Beta-naphthol represents a recalcitrant pollutant that when manipulated via polymerization, produces a polymer that has application in various industries^11^. This enzymatic approach reduces reliance on harsh chemicals and minimizes secondary pollution, making it a valuable strategy for the treatment of phenol-contaminated effluents^12^.

Dye-decolorizing peroxidases (DyPs) are a group of heme-containing enzymes that are mainly known for their ability to break down dyes through peroxidase activity. In recent years, studies have shown that their role is not limited to dye degradation, as they can oxidize a wide range of compounds, including carotenoids, phenols, and aromatic sulfides^13^. They have also proven to play a role in lignin degradation and in some cases, lignin synthesis^14,15^. The mechanism by which they catalyze polymerization reactions is based primarily on their ability to initiate free radical formation of a substrate which then proceeds to form a growing polymer. Being a recently discovered class of enzymes, not known until the late 90s, DyP-peroxidases have been under continuous investigation ever since. The microbial sources of this class of enzymes are diverse, expanding from white rot fungi to different actinomycetes^16–18^. The enzymes are classified into 4 main groups based on certain properties harbored by each of the different classes of enzymes^19^.

Pyrogallol (1,2,3-trihydroxybenzene) (PG) is a phenolic compound characterized by a benzene ring bearing three hydroxyl groups, which confer high chemical reactivity and strong reducing properties. It is a toxic compound. Exposure to this compound can cause irritation and cellular damage, which limits its direct applications^20^. To reduce its toxicity, PG can be converted into polymers through chemical or enzymatic oxidation^21^. Chemically, oxidants such as air, hydrogen peroxide, or metal ions convert PG into quinones, which then react to form brown to black polyphenolic polymers^22^. Enzymatically, oxidative enzymes like laccases, tyrosinases, and peroxidases catalyze the formation of semiquinones that spontaneously polymerize under mild aqueous conditions^23^. This polymerization not only reduces the compound’s toxicity but also produces polymers with antioxidant and antimicrobial properties^24^. PG and its polymers have applications in medicine, research, and industry, serving as antioxidants, precursors for resins, adhesives, and functional biomaterials; with the polymerization process enhancing safety and expanding potential uses^25^.

Limited evidence exists for DyP-mediated PG polymerization, while many studies demonstrate DyPs role in radical coupling and oligomer formation for other phenolics and lignin models, emphasizing their role in degradation and controlled polymerization^26–28^. An example includes the cross coupling of phenolic substrates such as catechin-catechol and catechin-guaiacol catalyzed by DyPs from *Streptomyces albidoflavus*^27^. In addition, DyP2 from *Amycolatopsis 75iv2* catalyzes the oxidation of O-glycosides into coupled oligomers with simultaneous radical-mediated bond cleavage^28^. Additionally, *Pp*DyP and its variants have been shown to promote the C-C coupling of lignin model compounds (such as divanillin and syringaresinol), forming high molecular weight dimers which can be used as polymer precursors or as scaffolds for fine chemicals^26^.

**Fig. 1:**
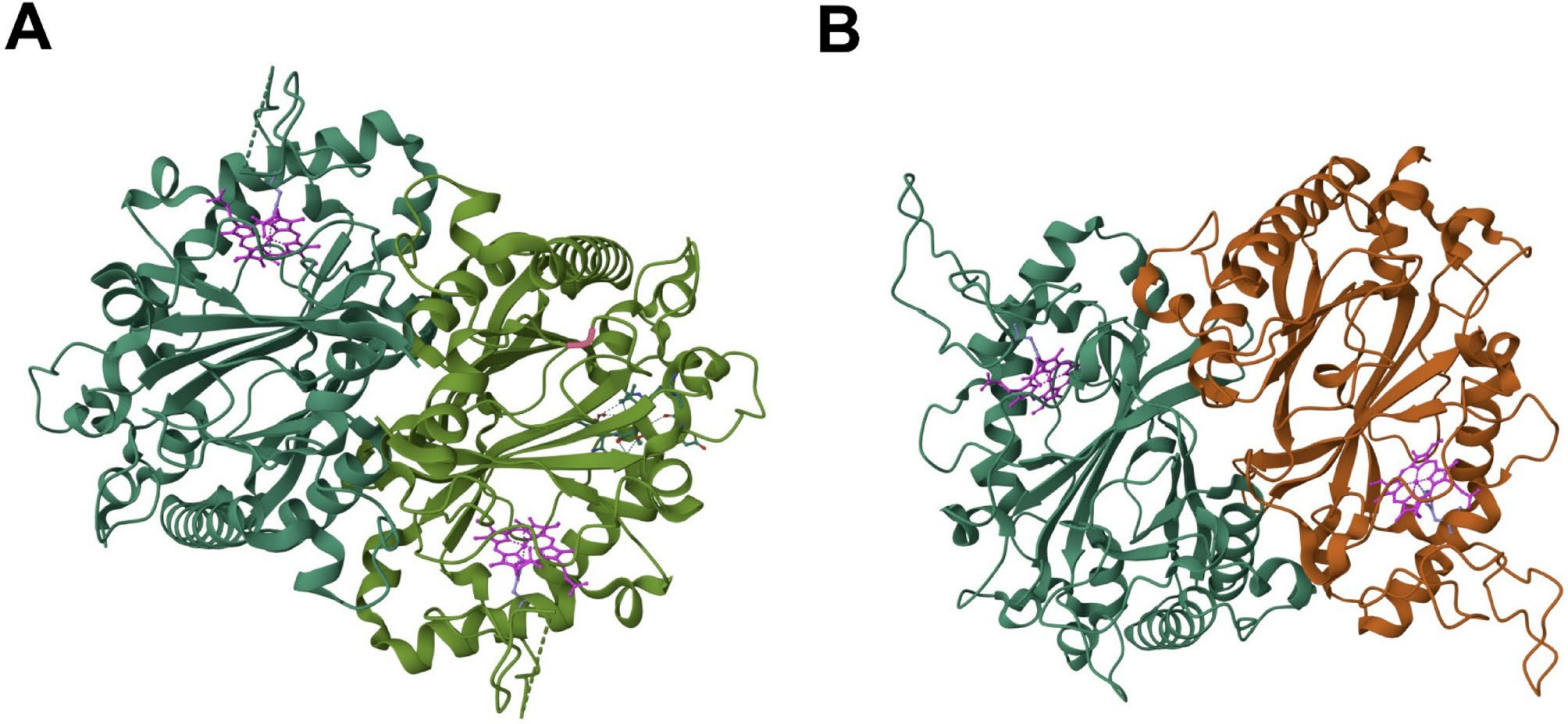
The crystal structures of recombinant DyP-peroxidases. (**A**) *Tfu*DyP (PDB ID 5FW4) and (**B**) *Cbo*DyP (PDB ID 6QZO) are represented via a cartoon representation showing the proteins as dimers each bearing a heme group (mauve) presented using the ball and stick representation.

In this work, we compare the effect of three members of the DyP-peroxidase family namely *Cbo*DyP from *Cellulomonas bogoriensis*^29^, *Tfu*DyP from *Thermobifida fusca*^30,31^, and *Svi*DyP from *Saccharomonospora viridis*^32^ for their ability to catalyze the polymerization of PG. *Tfu*DyP and *Cbo*DyP share key features of class A DyPs, including the Tat-signal sequence and a conserved histidine residue for heme binding (Fig. 1A and B). However, slight variations in a critical motif account for differences in activity. The polymerization efficiency of all three enzymes was evaluated by measuring the amount of polymer produced, allowing a direct comparison of their catalytic performance toward PG transformation.

## Results and discussion

### Enzyme expression and purification

**Fig. 2:**
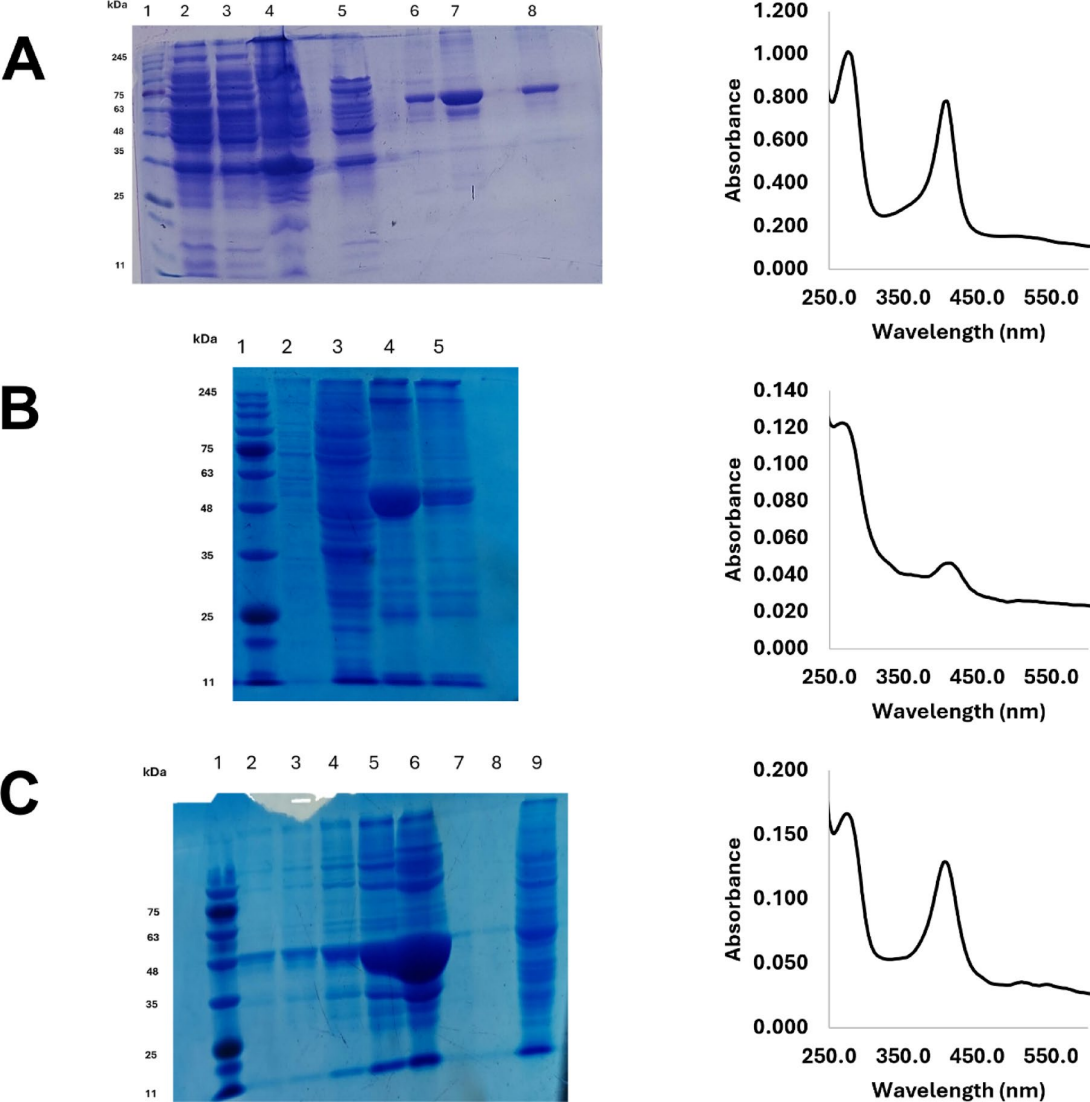
Protein gels showing the different fractions from purification (left) and a UV-Vis spectrum focusing on the peaks at 280 nm and 410 nm (soret) (right) of (**A**) *Cbo*DyP, (**B**) *Tfu*DyP and (**C**) *Svi*DyP from expressed cultures. Purification fractions are as follows: (**A**) 1 – Protein ladder, 2 – Crude lysate, 3 – Cell free extract, 4 – Pellet, 5 – Flowthrough, 6 – Wash from low imidazole buffer, 7 – Eluted protein, 8 – Protein after desalting in potassium phosphate buffer. (**B**) 1 – Protein ladder, 2 – Cell free extract, 3 – Flowthrough, 4 & 5 –Eluted protein. (**C**) 1 – Protein ladder, 2 – Eluted protein Frac 5, 3 – Eluted protein Frac 4, 4 – Eluted protein Frac 3, 5 – Eluted protein Frac 2, 6 – Eluted protein Frac 1, 7 – Wash from low imidazole buffer, 8 – Wash from potassium phosphate buffer, 9 – Flowthrough.

The 3 enzymes, *Cbo*DyP, *Tfu*DyP, and *Svi*DyP, produced distinct bands at the correct sizes in the Ni-column eluants from high-imidazole buffer. The *Cbo*DyP eluted fraction (Lane 8, Fig. 2A) was relatively pure showing a band at 54 kDa. The *Tfu*DyP fractions eluted from the Ni-column using high imidazole buffer show some impurities, but a distinct and evident band can be seen at ≈ 48 kDa close to its calculated molecular weight at 46 kDa (Lane 4 & 5, Fig. 2B). *Svi*DyP shows a relatively pure band at ≈ 48 kDa also indicating decent purification levels of the enzyme close to its calculated molecular weight at 44 kDa (Lane 2 & 3, Fig. 2C).

### Heme incorporation

The UV-Vis spectra (Fig. 2 – right) revealed various heme-loading behavior for each of the 3 DyP-peroxidases. *Cbo*DyP and *Svi*DyP showed a Reinheitszahl (Rz value) of 0.78 and 0.79, respectively indicating that more than 75% of the expressed protein was loaded with heme and thus was active. *Tfu*DyP on the other hand had a much lower Rz value approaching 0.4.

### Polymer synthesis

The polymerization of PG by the expressed DyP-type peroxidases (*Cbo*DyP, *Tfu*DyP, and *Svi*DyP) was evaluated through a series of optimized reaction conditions. Initially, the reaction was carried out using 5 mM PG in 50 mM potassium phosphate buffer at pH 6.5 with 1 µM enzyme, however, the yield was low. Lowering the pH to 5 improved the yield; however, incubation at 40 °C for 24 h still resulted in insufficient polymer formation. This comes in line with former reports showing enhanced activity upon lowering the pH levels to more acidic ranges. *Svi*DyP was found to experience a broad pH adaptability from pH 4–9 (> 35% activity) retaining 62% of its initial activity in buffers ranging from a pH of 6–8^32^. *Tfu*DyP was shown to display optimal activity at a pH of 3.5 when tested with Reactive Blue 19 as a substrate^30^. Using the same substrate, we formerly assessed the activity and found it to be optimal at pH 5 for *Cbo*DyP^29^. To enhance the yield, the reaction conditions were further modified by maintaining the initial incubation at 40 °C for 24 h, followed by continued incubation at 37 °C with shaking at 180 rpm for 3 days. In this optimized setup, hydrogen peroxide was added to a final concentration of 100 µM, and the enzyme concentration was maintained at 1 µM. This adjustment significantly improved polymerization efficiency. The reaction was subsequently terminated by heating at 100 °C for 2 days to deactivate the enzyme and promote buffer evaporation, resulting in the recovery of solid polymer.

**Fig. 3:**
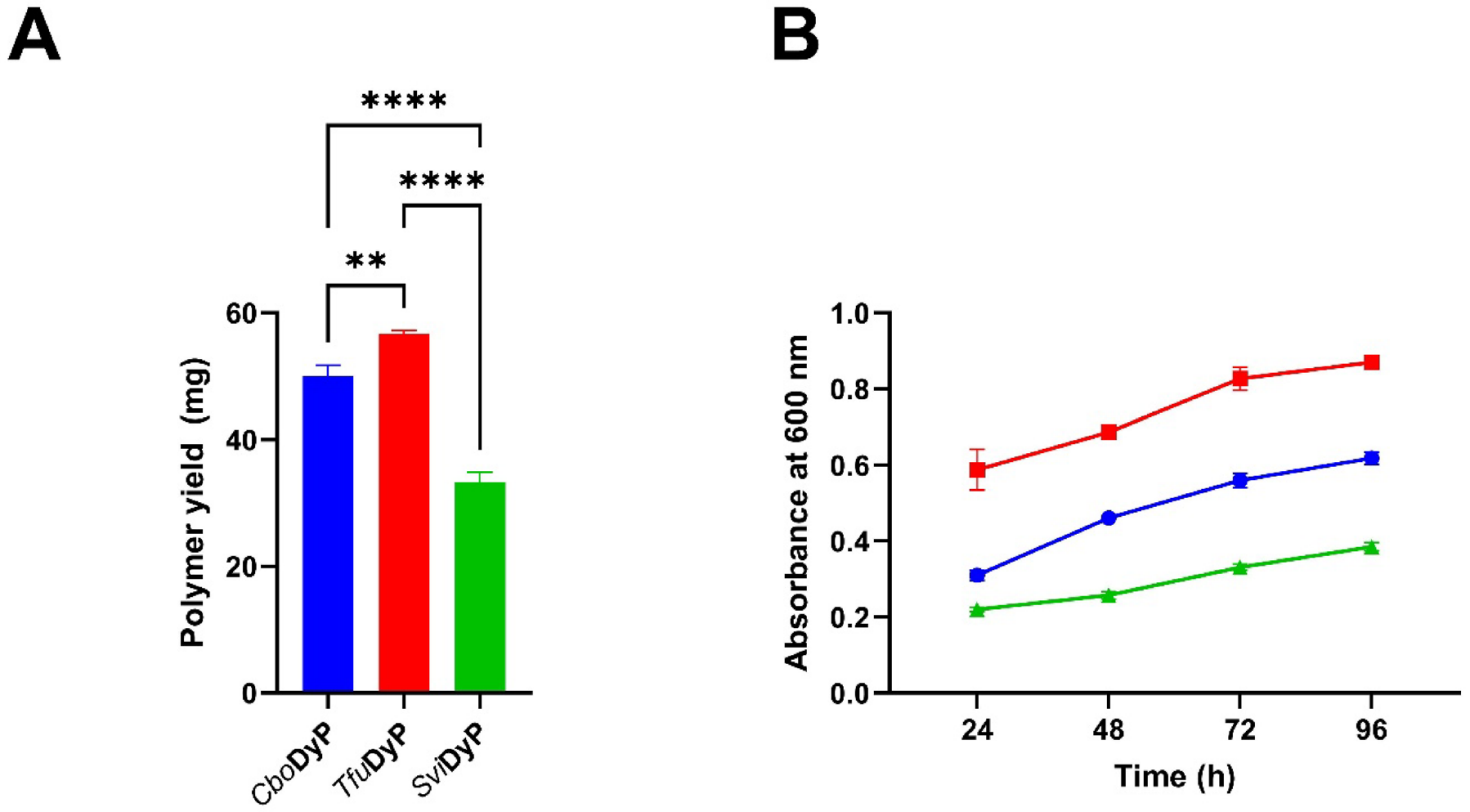
Monitoring PPG formation. (**A**) Yield in mg of PPG produced from 100 mg PG and (**B**) time course of increase in absorbance at 600 nm relative to color intensity from polymer formation. *Cbo*DyP (blue), *Tfu*DyP (red) and *Svi*DyP (green) were added at a starting concentration of 1 micromolar in both reactions.

The three enzymes showed significant differences in the amount of polymer they produced from PG. In a 100 mg-scale conversion, *Tfu*DyP, *Cbo*DyP and *Svi*DyP yielded 57 ± 0.5 mg, 50 ± 1.7 mg and 33 ± 1.5 mg of polymer, respectively (Fig. 3A). Statistical analysis was performed using GraphPad Prism. All produced polymer samples were insoluble in dimethyl sulfoxide (DMSO), dimethyl formamide (DMF), tetrahydrofuran (THF) and chloroform, as depicted in Fig. S1 in the supplementary material section, which aligns with their rigid molecular structure, strong intermolecular interactions, and high chain packing efficiency. Furthermore, elemental analysis (C, H) showed values of 63% C and 6% H which is in good agreement with theoretical values based on the polymer repeat unit (i.e., 62.3% C, 6.49% H).

To give more accurate and specific results, we decided to normalize the yields of polymer generated from each enzyme in a 100-mg scale to the fraction of actual active enzyme. Using ABTS as a reference substrate, we measured the specific activity of each enzyme in U/mg using the same starting enzyme concentration (1 micromolar) and pyrogallol concentration (5 millimolar) as in the originally devised experiment. The average specific activities for each of *Cbo*DyP, *Svi*DyP and *Tfu*DyP were 5.56 ± 0.36, 1.74 ± 0.36 and 3.56 ± 0.49 U/mg, respectively. After normalizing the yields of polymer formation with respect to 1 U/mg specific activity for each enzyme, the yield of polymer formed for *Cbo*DyP, *Svi*DyP and *Tfu*DyP were 9, 19 and 16 mg, respectively. This shows that the maximum yield of polymer was formed with *Svi*DyP followed by *Tfu*DyP and finally *Cbo*DyP. The fact that *Svi*DyP gave the highest yield of polymer formation correlates nicely with the Rz value of the enzyme but the pattern is not similar with *Cbo*DyP despite its high Rz value. *Tfu*DyP on the other hand has a high polymer formation yield indicating that even at low heme loading, the enzyme retains a significant amount of its activity. Also, to validate that the process of polymerization proceeds over 96 h, we devised an experiment to follow up with the increase in absorbance at 600 nm as a result of an increase in color intensity in the reaction owing to polymer formation over time. The results showed a similar pattern to our initial experimental procedure when we based concentration on molarity giving the highest change in absorbance in the reaction catalyzed using *Tfu*DyP followed by *Cbo*DyP and finally *Svi*DyP (Fig. 3B) as we replicated the original conditions of the 1 micromolar initial enzyme concentration experiment. No residual enzyme activity could be detected after 96 h using a crude ABTS assay indicating that the polymerization reaction proceeds regardless of the presence of active enzyme in solution and that the enzyme is only needed to initiate the polymerization reaction. Lack of polymerization activity in the control run with a light brown color formed from pyrogallol oxidation using *E. coli* lysate passed through the Ni-Nta column assumes that activity seen with all 3 enzymes is due to the enzyme itself rather than the impurities formed from *E. coli* background proteins. The difference in activity between the 3 DyPs can be explained by combining both mechanistic and microbiological factors^33^. From a structural point of view, each enzyme has a slightly different active site, which affects how well PG can bind and be positioned for the reaction. A better fit inside the active site means more efficient oxidation and higher polymer production. Differences in the charge around the active site can also influence how stable the reaction intermediates are, which directly affects how smoothly the polymerization process continues^25^.

Microbiological factors play a significant role in polymerization efficiency as well. Since these enzymes originated from different microorganisms, they naturally differ in their stability and behavior under the same conditions^34^. In addition, because they were expressed from various *E. coli* expression host strains, variations in expression level, protein folding, and proper heme incorporation could affect how much active enzyme is available. Some enzymes may remain stable and active for longer periods, while others may lose activity more quickly due to sensitivity to hydrogen peroxide or reaction conditions^35^.

Overall, the variation in yield between the three enzymes is likely due to a combination of factors: how well PG binds, the charge environment inside the enzyme, and how efficiently each enzyme carries out the reaction. These small differences in structure and activity can lead to noticeable changes in the final amount of polymer produced^36^.

In addition, recombinant DyP-type peroxidases also have several practical advantages that make them more convenient, and controllable compared to conventional oxidoreductases such as fungal laccases for the oxidative polymerization of phenolic compounds^37^. Recombinant DyP-type peroxidases utilize hydrogen peroxide as an oxidant to initiate catalysis, enabling precise control of the reaction through modulation of hydrogen peroxide concentration^38^. In contrast, laccases depend on oxygen from the air, so their activity can be affected by how well oxygen dissolves in the solution and how the reaction is mixed, which can make results less consistent, especially when scaling up^39^. DyPs avoid this limitation because they do not rely on oxygen transfer, leading to more stable and uniform reaction conditions. However, particular consideration must be taken on the toxic effect of excessive use of hydrogen peroxide. Recombinant DyPs can be improved through protein engineering to make them more resistant to inactivation caused by hydrogen peroxide, which is a common issue in these enzymes^40^. Overall, this makes DyPs more reliable and easier to control for producing phenolic polymers with consistent properties compared to the laccase-based system.

### Polymer characterization

#### Morphology

**Fig. 4:**
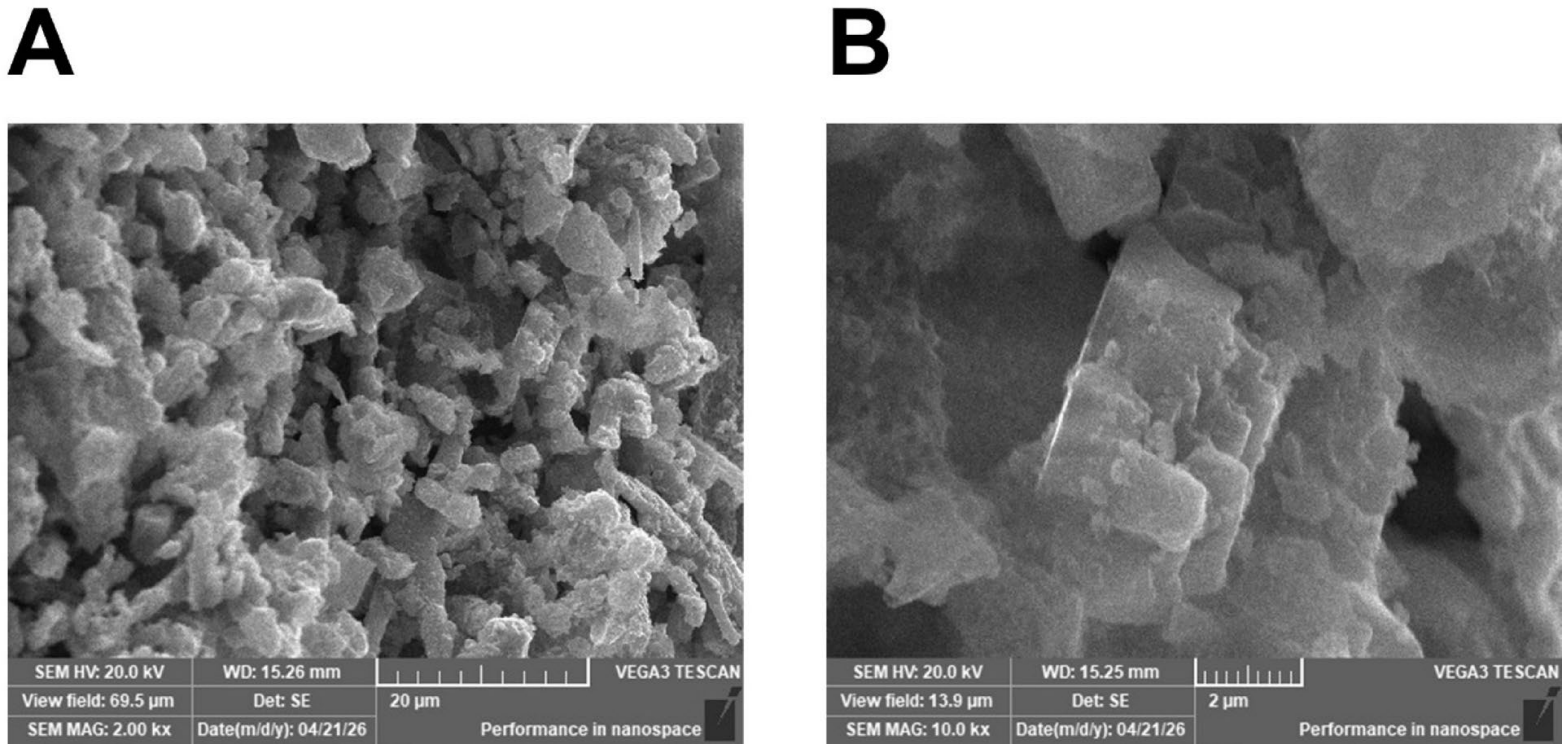
SEM micrographs of PPG polymer. The polymer is prepared using *Tfu*DyP at 2.00 kx (**A**) and 10.0 kx (**B**) magnification.

The surface morphology of the synthesized PPG was examined using scanning electron microscopy (SEM) (Fig. 4). At low magnification, the material exhibits a heterogeneous and aggregated morphology composed of irregularly shaped clusters without well-defined geometric features (Fig. 4A)^41,42^. At higher magnification (Fig. 4B), the polymer surface appears rough and textured, with granular and nodular domains distributed throughout the sample. The absence of faceted crystalline structures and the presence of irregular aggregates suggest a material with limited long-range structural order^43^. These observations are consistent with the heterogeneous nature of oxidatively derived polyphenolic materials^43^. SEM analysis therefore provides morphological evidence for the formation of a solid polymeric product, while detailed structural information was obtained from the complementary FTIR, Raman spectroscopy, and XRD analyses discussed below.

Overall, the SEM analysis confirms that the synthesized PPG is characterized by a disordered, aggregated, and highly cross-linked morphology, consistent with an oxidative polymerization mechanism involving phenoxy radicals. The rough surface texture, lack of crystallinity, and presence of aggregated domains collectively support the formation of a heterogeneous polymeric network with potential functional surface properties.

### Fourier transform infrared spectroscopy

**Fig. 5:**
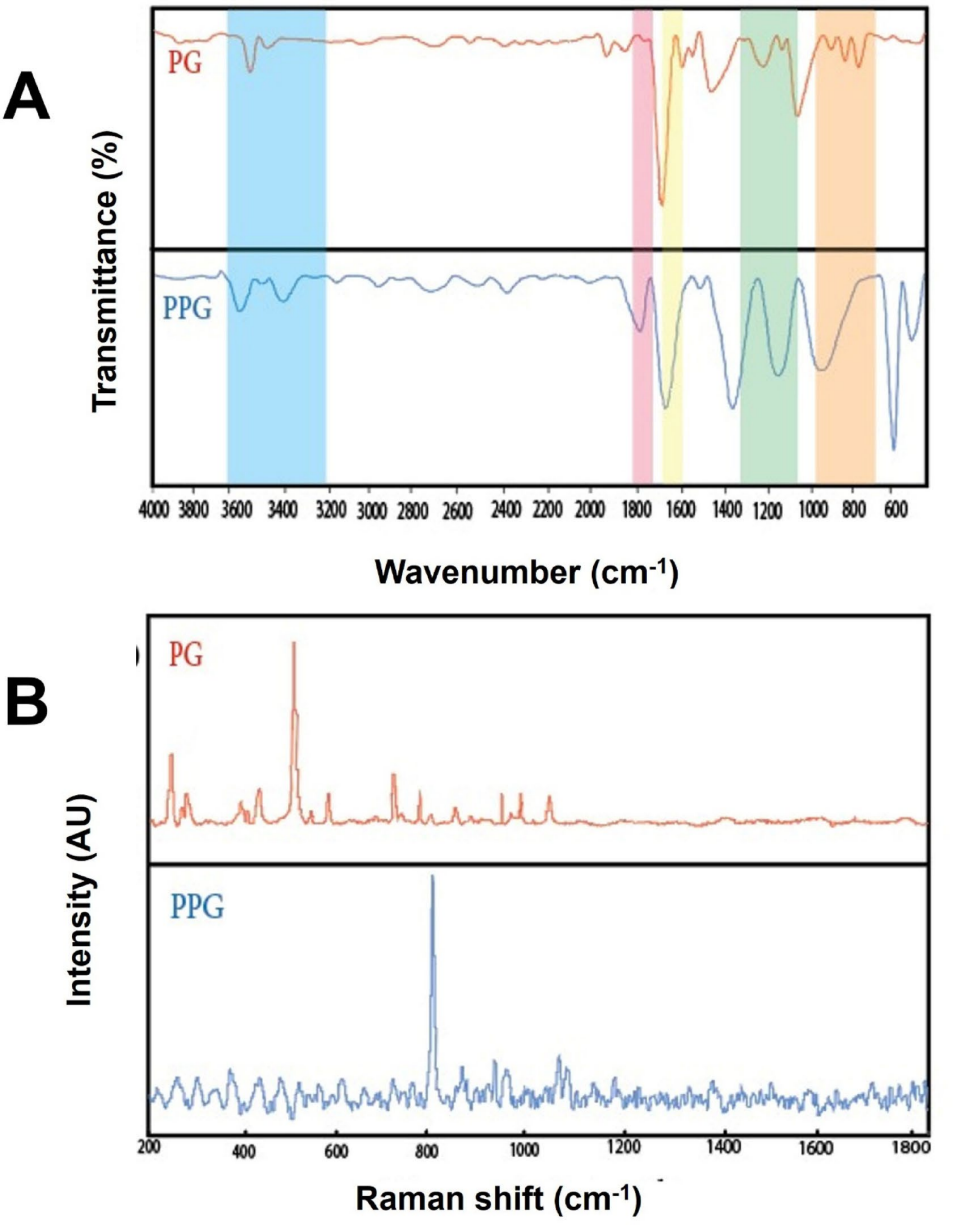
FTIR and Raman spectra for PG and PPG. (**A**) FTIR spectra for PG (red) and PPG (blue). Highlighted regions correspond to: O–H stretching vibrations (3200–3600 cm^−1^) (light blue), C = O stretching of quinone-like structures (1650–1720 cm^−1^) (rose), aromatic C = C stretching vibrations (1580–1620 cm^−1^) (yellow), C–O–C stretching vibrations (1000–1300 cm^−1^) (green), and aromatic out-of-plane C–H bending vibrations (600–900 cm^−1^) (orange). (**B**) Raman spectra for PG (red) and PPG (blue). The PPG polymer was prepared using 1 micromolar *Tfu*DyP and 100 mg PG as a starting substrate.

The FTIR spectrum of PG (1,2,3-trihydroxybenzene) monomer, exhibited characteristic absorption bands typical of phenolic aromatic compounds (Fig. 5A). A broad band observed in the 3200–3500 cm⁻¹ region is attributed to O–H stretching vibrations, associated with extensive hydrogen bonding among the hydroxyl groups, confirming the presence of phenolic –OH functionalities^44^. The band at approximately 1600–1620 cm⁻¹ corresponds to the aromatic C = C skeletal vibrations of the benzene ring. Additionally, bands in the range of 1200–1350 cm⁻¹ are assigned to C–O stretching of phenolic groups. The peaks appearing between 750 and 900 cm⁻¹ arise from C–H out-of-plane bending modes, indicative of a substituted aromatic ring structure^44,45^.

Following enzymatic polymerization, the FTIR spectrum of PPG reveals significant structural changes, consistent with oxidative coupling and the formation of a conjugated, cross-linked polymeric network. The O–H stretching region (3200–3600 cm⁻¹) (blue shaded area) becomes broader and slightly shifted, reflecting enhanced inter- and intra-molecular hydrogen bonding, likely due to the coexistence of residual phenolic groups and newly formed hydroxyl/quinone interactions^46^. The appearance or intensification of a band in the range of 1650–1720 cm⁻¹ (pink shaded area) is assigned to C = O stretching vibrations of quinone-like structures, providing strong evidence for oxidative transformation during polymerization^47^.

Moreover, the band near 1580–1620 cm⁻¹ (yellow shaded area) becomes broader and less defined compared to the monomer, corresponding to aromatic C = C stretching and indicating increased π-conjugation alongside a loss of structural symmetry due to random coupling and cross-linking^23,24^. The prominent band observed between 1000 and 1300 cm⁻¹ (green shaded area) is attributed to C–O–C stretching vibrations, suggesting the formation of ether linkages within the polymer backbone^23,24,46,47^. In addition, the low wavenumber region (600–900 cm⁻¹) (orange shaded area) exhibits increased intensity and spectral complexity, associated with aromatic substitution patterns and out-of-plane C–H bending modes, further confirming the heterogeneous and disordered nature of the polymer structure^24^.

### Raman spectroscopy

The Raman spectra of PG monomer and PPG obtained via peroxidase-mediated enzymatic polymerization are presented in Fig. 5B. The spectra revealed pronounced differences in peak intensity, sharpness, and distribution, reflecting the structural transformation from a well-defined molecular system of PG to a disordered polymeric network of PPG.

The Raman spectrum of PG exhibited sharp and well-resolved peaks, characteristic of a highly ordered aromatic compound. The most prominent feature appeared around ~ 550–600 cm⁻¹, which can be attributed to aromatic ring deformation and C–O stretching vibrations associated with phenolic groups. Additional bands observed in the ~ 300–500 cm⁻¹ region corresponded to out-of-plane ring bending modes, reflecting the substituted benzene structure. In the higher wavenumber region, bands between ~ 800–1100 cm⁻¹ were assigned to C–H in-plane bending and C–O stretching vibrations, while weaker features extending toward ~ 1200–1600 cm⁻¹ correspond to aromatic C = C stretching modes. The sharpness and intensity of these peaks indicate a well-defined molecular structure with limited structural disorder.

In contrast, the Raman spectrum of PPG showed significant broadening and loss of peak definition, indicative of increased structural disorder and heterogeneity following polymerization. The most prominent feature was a strong band centered around ~ 750–800 cm⁻¹, which is associated with aromatic ring breathing modes and C–O–C linkages, suggesting the formation of ether-type bonds during oxidative coupling.

The spectral region between ~ 1200–1600 cm⁻¹ became broader and less distinct compared to the monomer, reflecting overlapping vibrational modes of conjugated aromatic systems. This broadening is characteristic of extended π-conjugation and random cross-linking, resulting in a distribution of bonding environments rather than discrete molecular vibrations. Additionally, the overall reduction in peak sharpness and the emergence of a more continuous spectral profile indicates the formation of an amorphous, cross-linked polymeric network. This behavior is consistent with the generation of phenoxy radicals during enzymatic oxidation, compatible with the formation of a structurally heterogeneous polyphenolic material.

Overall, the Raman spectra provided strong evidence for the successful enzymatic polymerization of PG. The transformation from a well-defined monomeric structure to a disordered, conjugated polymer was confirmed by the broadening of vibrational bands, loss of spectral resolution, and the emergence of features associated with cross-linked aromatic networks. These findings are in excellent agreement with the proposed oxidative coupling mechanism and support the formation of a structurally complex PPG material.

### Powder X-ray diffraction

**Fig. 6:**
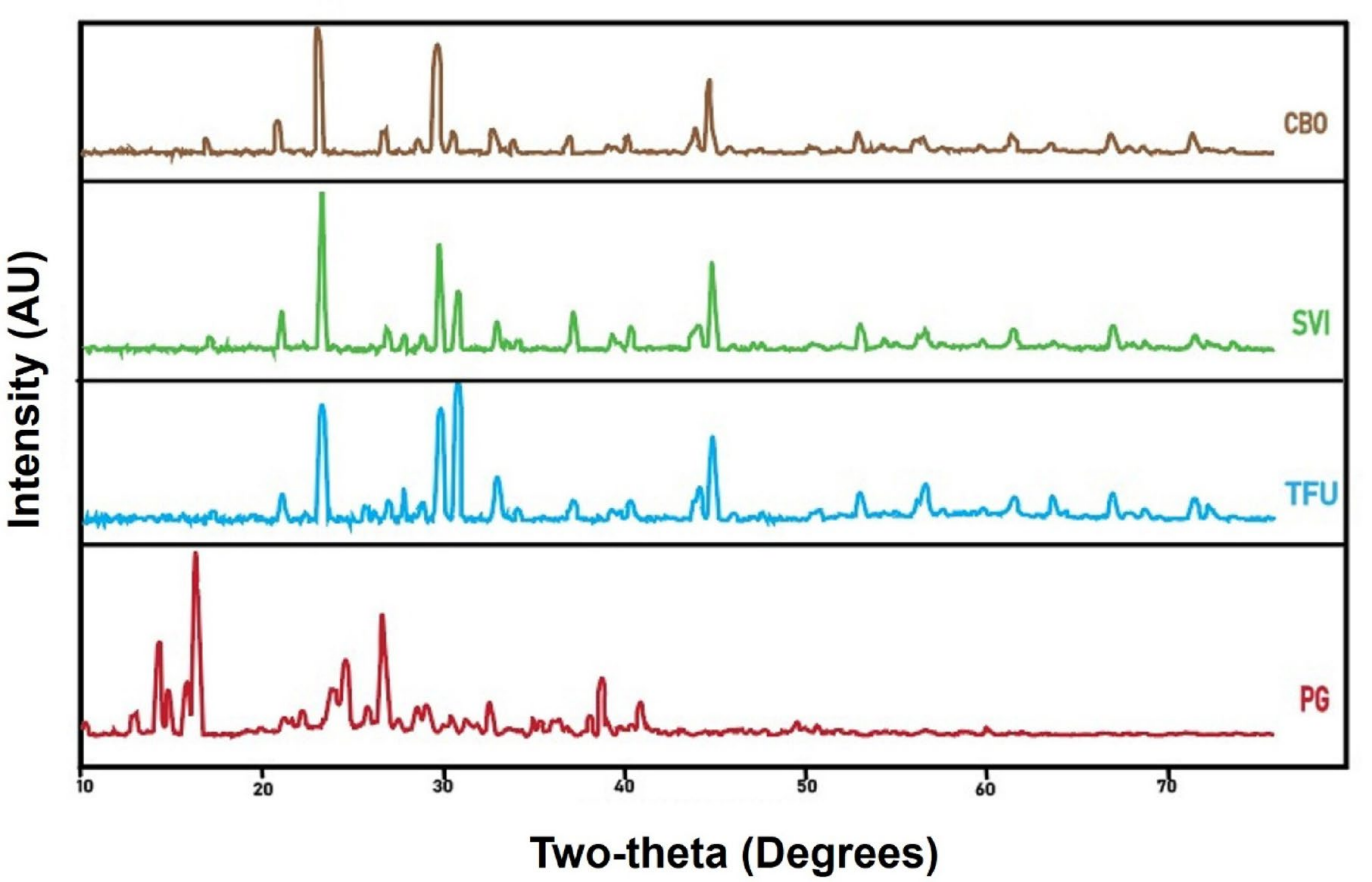
XRD patterns of PG monomer (red) and PPG polymers. The reactions were prepared using 1 micromolar of each of *Tfu*DyP (cyan), *Svi*DyP (green) and *Cbo*DyP (brown).

The X-ray diffraction (XRD) patterns of PG monomer and PPG polymers synthesized via peroxidase-mediated enzymatic polymerization using different enzyme sources; *Tfu*DyP, *Svi*DyP and *Cbo*DyP are presented in Fig. 6.

The XRD pattern of PG exhibited multiple sharp and intense diffraction peaks, indicating a highly crystalline structure. The well-defined reflections observed in the low to mid 2θ range (approximately 10–40°) are characteristic of a molecular crystal with long-range order, arising from the regular packing of PG molecules through intermolecular interactions such as hydrogen bonding and π–π stacking. The presence of numerous distinct peaks confirms that PG exists in a well-organized lattice, consistent with its small molecular structure and ability to form ordered crystalline domains.

In contrast, the XRD patterns of PPG (*Tfu*DyP, *Svi*DyP and *Cbo*DyP) displayed significant changes in peak shape, intensity, and distribution, reflecting the transformation from a crystalline monomer to a less ordered polymeric material. Compared to PG, the PPG samples showed reduced peak intensity, broadening of diffraction peaks and partial loss of sharp reflections. These features indicate a marked decrease in crystallinity, suggesting that the polymerization process disrupts the regular packing of PG molecules. The formation of covalent bonds between monomer units results in a three-dimensional, cross-linked network, which prevents the establishment of long-range order. The PPG patterns exhibited a combination of broad halos (indicative of amorphous regions) and residual sharp peaks (indicative of short-range ordering). This suggests that PPG possesses a semi-crystalline nature, where amorphous domains arise from random cross-linking and localized ordering may persist due to π–π interactions between aromatic segments. Overall, all PPG samples showed similar overall trends, subtle differences can be observed. The XRD results are in strong agreement with FTIR analysis, which indicates the formation of new functional groups (e.g., quinone, ether linkages), Raman spectroscopy, showing increased structural disorder and conjugation, and SEM observations, revealing heterogeneous and aggregated morphology. Together, these findings consistently confirm the formation of disordered, cross-linked polymeric material.

In summary, XRD patterns revealed a clear transition from the highly crystalline structure of PG to a predominantly amorphous or semi-crystalline PPG, characterized by peak broadening, reduced intensity, and loss of long-range order, consistent with the formation of a cross-linked polymeric network via oxidative enzymatic polymerization.

## Materials and methods

### Plasmid transformation, protein expression, and purification

Plasmids, each carrying a single gene for one of the three enzymes (*Cbo*DyP, *Tfu*DyP and *Svi*DyP) (GECCO-Biotech, Groningen, The Netherlands), were individually transformed into *E. coli* TOP10 competent cells. This resulted in three separate transformants, each containing one plasmid. The transformed cells were plated onto LB agar (Sigma Aldrich, Burlington, MA, USA) plates containing 100 µg mL^− 1^ ampicillin (Sigma Aldrich, Burlington, MA, USA)^29^. An overnight pre-culture (5 mL) in LB broth (Sigma Aldrich, Burlington, MA, USA) was inoculated using one of the isolated colonies from each type of clone and the medium supplemented with 100 µg mL^− 1^ ampicillin. The pre-culture was used to inoculate a 500 mL terrific broth (TB) (Sigma Aldrich, Burlington, MA, USA) medium and ampicillin was added to give 100 µg mL^− 1^ as a final concentration in the medium. The culture grew at 37 °C. Once an OD (optical density) of 0.6 was reached, an amount equivalent to 0.02% (v/v) L-arabinose (Sigma Aldrich, Burlington, MA, USA) was added to the culture to induce protein expression. The flask was then transferred to an incubator operating at 24 °C and 180 rpm for *Cbo*DyP, and 30 °C and 180 rpm for *Tfu*DyP and *Svi*DyP.

### Harvesting and purifying the expressed peroxidases

The cultures were harvested after 24 h by centrifugation at 6000× *g* for 20 min at 4 °C (Centurion Scientific, West Sussex, UK). The pellet was disrupted by sonication (10 s on, 10 s off for 1 h at 100% amplitude) using a Branson sonicator (Brookfield, CT, USA). The sonicated cells were centrifuged for 30 min at 6,000 x *g* to separate the cell debris from the cell-free extract. The cell free extract was added to a prepacked Cytiva 1 mL Ni-NTA cartridge (Marloborough, MA, USA) after equilibrating the resin using 10 column volumes of potassium phosphate 50 mM, pH 8.0 containing 500 mM NaCl and 5% glycerol (v/v) (Sigma Aldrich, Burlington, MA, USA). The column was washed using 5 column volumes of 50 mM potassium phosphate, pH 8.0, containing 500 mM NaCl, 5% glycerol, and 10 mM imidazole. The enzyme was then eluted using 50 mM potassium phosphate, pH 8.0 containing 500 mM NaCl, 5% glycerol, and 500 mM imidazole. The protein was desalted using a G25 Desalting Purification Cartridge (Biocomma, Guangdong, China) using 50 mM potassium phosphate pH 7.5 with 150 mM NaCl, and 10% glycerol (v/v). The different fractions from the protein purification were visualized on protein gel after SDS-PAGE.

### Determination of enzyme concentration

The concentrations of the three enzymes were estimated spectrophotometrically using their predicted molar extinction coefficients at 280 nm determined using the Protparam Expasy Tool^48^. The values were 62.575 mM⁻¹ cm⁻¹ for *Cbo*DyP, 48.595 mM⁻¹ cm⁻¹ for *Svi*DyP, and 45.950 mM⁻¹ cm⁻¹ for *Tfu*DyP.

### UV-Vis spectrum scan

*Cbo*DyP, *Tfu*DyP and *Svi*DyP spectrum scan was done in potassium phosphate buffer 50 mM at pH 7.5 using a UV-Vis Spectrophotometer single beam device (Peak Instruments, USA). Fifty microliters of each crude enzyme was added to a quartz cuvette containing 950 µl of 50 mM potassium phosphate buffer. Then the device was adjusted to perform a wavelength scan in the range 190–900 nm.

### Stepwise optimization of pyrogallol polymerization

The polymerization ability of the expressed DyP-type peroxidases (*Cbo*DyP, *Tfu*DyP, and *Svi*DyP) toward PG (Techno PharmChem, India) was evaluated by incubating 5 mM PG in 50 mM potassium phosphate buffer pH 6.5 initially, then lowered to pH 5. Hydrogen peroxide was then added to achieve a final concentration of 100 µM, followed by the addition of the enzyme to a final concentration of 1 µM. The reaction mixture was incubated initially at 40 °C for 24 h, then optimized to include an additional incubation at 37 °C with shaking at 180 rpm for 3 days. The reaction was terminated by heating the solution at 100 °C in a B5050 F Heraeus (Hanau, Germany) oven for 2 days to deactivate the enzyme and promote buffer evaporation, resulting in the recovery of solid polymer.

A similar experiment was done to monitor the polymer formation over time. Polymer formation was monitored spectrophotometrically by measuring the optical density (OD) of the reaction mixture after 24, 36, 72, and 96 h of incubation.

### Conversion 100-milligram scale

A 100-milligram scale conversion of PG was carried out in a final volume of 50 mL in a 250 mL screw-cap bottle. The reaction mixture contained 100 mg PG and the rest of the conditions were maintained according to optimum conditions mentioned earlier. The experiment was repeated three times^11^. The polymer was analyzed using different tools including SEM, FTIR, Raman Spectroscopy, and XRD.

### Determination of specific activity using ABTS as a reference substrate

To mimic the conditions for the pyrogallol conversion, 5 mM of ABTS and 100 µM hydrogen peroxide were added to sodium citrate 50 mM pH 4 buffer in a cuvette. 1 µM of each enzyme was then added in separate experiments to initiate the reaction and the activity followed for 60 s at 415 nm using the kinetics program on a UV-Vis Spectrophotometer (Peak Instruments). The initial rate was determined and used to calculate the specific activity at the tested conditions.

### Scanning electron microscopy (SEM)

Scanning electron microscopy (SEM) micrographs were obtained using a Lyra3 GMU FIB-SEM system (Tescan, Brno, Czech Republic). The PPG samples were mounted onto aluminum stubs using double-sided carbon adhesive tape and subsequently sputter-coated with a thin layer of gold/palladium prior to imaging.

### Fourier transform infrared spectroscopy (FT-IR)

Fourier transform infrared (FTIR) spectra were acquired in attenuated total reflection (ATR) mode using a Nicolet iS10 spectrometer (Thermo Fisher Scientific, Waltham, MA, USA). The spectra were collected over the wavenumber range of 400–4000 cm⁻¹ at a spectral resolution of 4 cm⁻¹, with 30 scans averaged for each measurement.

### Raman spectroscopy

Raman spectra were acquired using an AFM-Raman microscope (NTEGRA Spectra, NT-MDT Spectrum Instruments, Moscow, Russia) equipped with a 10× objective and a 500 nm excitation laser. Measurements were performed over a Raman shift range of 200–2000 cm⁻¹. Each spectrum represents an average of ten individual scans, collected at a laser power of 1 mW with an acquisition time of 1 min per scan, to enhance the signal-to-noise ratio.

### X-ray diffraction (XRD)

X-ray diffraction (XRD) patterns were recorded using a Bruker D8 Advance diffractometer equipped with Cu Kα radiation (λ = 1.5418 Å), operated at 40 kV and 40 mA. Measurements were conducted over a 2θ range of 10–70°, with a step size of 0.02° and a scanning rate of 1° min⁻¹.

## Conclusion

In this work, we successfully managed to polymerize PG into PPG using three different DyP type peroxidases; *Tfu*DyP, *Cbo*DyP and *Svi*DyP. The polymer formed was characterized in detail using SEM, FTIR, Raman spectroscopy, XRD and elemental analysis revealing details characteristic of an amorphous polymer. The formed polymer could find applications in the adhesives and coatings industry among other biomedical fields. Future work could involve fine-tuning via experimental design optimization to open the way to generate polymers with properties needed for different or more tailored applications.

## Supplementary Information

Below is the link to the electronic supplementary material.

Supplementary Material 1

## Data Availability

All data supporting the findings of this study are available within the paper and its Supplementary Information.
