# Supplementary material for: Recombinant DyP peroxidases for green synthesis and characterization of poly(pyrogallol)
Source: Sci Rep. 2026 Aug 5;16:24139. doi: 10.1038/s41598-026-64069-8 (PMC13443537; doi:10.1038/s41598-026-64069-8)
Supplement: Supplementary file 1 [file 41598_2026_64069_MOESM1_ESM.docx]

**Recombinant DyP Peroxidases for Green Synthesis and Characterization of Poly(pyrogallol)**

Marina Refaat^1±^, Khaled O. Sebakhy^2±^, Menna-t-Allah M. Saad^1^, Marwa ElRakaiby^1^, Mohamed H. Habib^1,3*^ and Maha M. Ismail^1^

^1^Department of Microbiology and Immunology, Faculty of Pharmacy Cairo University, 11562 Kasr El-Aini, Cairo, Egypt

^2^Chemical and Industrial Processes Engineering, Faculty of Engineering, Bahrain Polytechnic, Isa Town, The Kingdom of Bahrain

^3^Department of Microbiology and Immunology, Faculty of Pharmacy, Egypt-Japan University of Science and Technology (EJUST), New Borg El-Arab City, 21934 Alexandria, Egypt

Supporting Information

**Figure S1.** **The solubility of PPG synthesized from left to right in each image using 1 micromolar *Svi*DyP, *Cbo*DyP and *Tfu*DyP starting with 100 mg PG** – (A) solubility in water, (B) solubility in DMSO, (C) solubility in DMF, (D) solubility in Chloroform and (E) solubility in THF
